# A TALE of *SWEET* genes: Transcriptomic analysis reveals the first black rot susceptibility genes in cauliflower

**DOI:** 10.1093/plphys/kiag768

**Published:** 2026-10-15

**Authors:** William J W Thomas

**Affiliations:** Assisstant Features Editor, Plant Physiology, American Society of Plant Biologists, Rockville, MD, United States; School of Biological Sciences, University of Western Australia, Perth, Western Australia, Australia

*Brassica oleracea* is an important vegetable crop cultivated globally. Selection for different traits in *B. oleracea* has resulted in impressive morphotypic diversity, and these morphotypes account for numerous household vegetables such as broccoli, Brussels sprouts, cabbage, cauliflower, kale, and kohlrabi. However, these vegetables are the hosts of several pathogens that cause yield-limiting disease. Among them, black rot, caused by the bacterial pathogen *Xanthomonas campestris* pv. *campestris* (*Xcc*), is one of the most devastating, resulting in up to 60% yield loss in susceptible cultivars (Greer et al 2023).

To boost their chance of successful infection, phytopathogenic bacteria have evolved sophisticated molecular mechanisms. *Xcc* deploys a range of effector proteins, which are virulence factors designed to manipulate host physiology or interfere with host immunity. While many *Xcc* effectors bind to immune-related proteins, some belong to the unique Transcription Activator-Like Effector (TALE) class and operate differently. TALEs make their way into the host cell’s nucleus and act as pathogen-encoded transcription factors. Here, they bind to specific DNA sequences, usually in a promoter region, to modulate the expression of genes, which in turn provides some benefit to the invading pathogen. The *Sugars Will Eventually be Exported Transporters* (*SWEET*) gene family is a known target for *Xanthomonas* TALEs. For example, in rice, *SWEET* genes are upregulated via TALE-mediated transcriptional modification, and this upregulation is essential for disease establishment by *X. oryzae* pv. *oryzae* (Oliva et al 2019). Similar interactions between *Xanthomonas* TALEs and *SWEET* genes have been reported in other crops, indicating that these interactions may be a conserved molecular strategy employed across *Xanthomonas* pathogens. Previous studies have shown that TALE-encoding genes in *Xcc* can be associated with pathogenicity in cauliflower (*B. oleracea* var. *botrytis*) (Denancé et al 2018; Zárate-Chaves et al 2023). However, what genes they are targeting in cauliflower and why is largely unknown.

Host genes that facilitate pathogen colonization, including the direct targets of TALEs, are often referred to as susceptibility (*S*) genes (Koseoglou et al 2022). Researchers have demonstrated that by mutating the *S* genes that pathogens hijack, they can reverse engineer resistance. For example, Tao et al (2021) used CRISPR/Cas9 to generate rice plants with knockout mutations in 3 different *S* genes, and these plants displayed enhanced resistance to rice blast and bacterial blight. Importantly, the key agronomic traits of these mutants were comparable to their unedited counterparts. Utilizing *S* genes is particularly promising in pathosystems in which there is little or no naturally occurring resistance in the host. This is the case for black rot in *B. oleracea*, in which complete resistance is exceedingly rare (Kong et al 2021).

Recently in *Plant Physiology*, Charleux et al (2026) investigated the role of a specific *Xcc* TALE in controlling the virulence of black rot in cauliflower. They chose the TALE Tal12a, which was identified as being one of the most widespread among *Xcc* strains in a previously studied diversity panel (Denancé et al 2018). To first validate the role of the TALE-encoding gene *tal12a* in isolation from other effectors, the authors took a two-pronged approach, performing loss-of-function and gain-of-function experiments. Mutant strains lacking the *tal12a* gene displayed significantly less chlorosis and necrosis on cauliflower leaves after 7 d postinoculation compared with the wild-type (WT). Conversely, when *tal12a* was stably integrated into a genetically engineered strain that lacks all known effectors, called Δ26, it partially restored virulence and increased disease severity compared with the WT. Together, these results confirm that *tal12a* encodes a TALE that contributes to *Xcc* virulence.

To explore the potential targets of Tal12a in the host, Charleux et al (2026) carried out RNA sequencing on infected cauliflower leaves at 1 d postinoculation. Two strains were used, the avirulent Δ26 and Δ26 complemented with *tal12a*. Any differential expression detected would therefore be attributed to the presence of Tal12a. The authors identified 380 cauliflower genes that were upregulated when using the complemented strain. They performed a similar experiment using another TALE, Tal14c, to determine if any of the same genes were upregulated. Only 43 genes were induced by both TALEs, and these were enriched for defense-related Gene Ontology terms. The authors suggest that this overlap may indicate that there is a common downstream pathway activated by different TALEs, despite having different DNA-binding specificities and therefore different target genes. To narrow down the 380 upregulated genes, Charleux et al (2026) also identified effector binding elements (EBEs) using two prediction tools, TALVEZ (Pérez-Quintero et al 2013) and PrediTALE (Erkes et al 2019). This analysis revealed 29 genes that contained high-confidence EBEs while also being upregulated. These genes were further refined through the characterization of features typical of direct TALE targets, including proximity to core regulatory elements such as the TATA box. Because the manipulation of *SWEET* genes has been demonstrated to facilitate *Xanthomonas* colonization, the authors also included 2 candidate *SWEET* genes that were highly upregulated, despite not having high-confidence EBEs detected within their promoters. A final list of 9 candidates was prioritized for further analysis.

To ensure that the induction of their 9 candidates was a result of TALE-dependent transcriptional regulation, the authors used CRISPR interference to disrupt the conserved ribosome binding site, which is common in all *tal* genes, in 2 *Xcc* strains. Through comparative expression analysis with the unedited WT, they confirmed that all 9 candidates were upregulated in a TALE-dependent manner. Logically, the next question to ask was: does Tal12a bind directly to the promoters of these 9 candidates in order to upregulate them? To answer this question, the authors carried out transactivation assays in *Nicotiana benthamiana* in which they fused the promoter region of each candidate to a promoterless reporter gene and codelivered these fusions together with a Tal12a-containing *Xcc* strain. These assays revealed that Tal12a directly regulates 7 of the candidates by binding to their promoters. However, they also uncovered that Tal12a did not directly regulate the remaining 2 candidates, *BoSWEET13* and *BoSWEET14c*, consistent with the lack of predicted EBE in their promoters.

Finally, to test the individual contribution of 3 promising candidates (*BoIAA7c*, *BoSWEET13*, and *BoSWEET14*) to *Xcc* virulence, Charleux et al (2026) constructed artificial TALEs (arTALEs) engineered to target and activate their promoters. The transcript levels of these genes after arTALE activation were comparable to or higher than when induced by Tal12a. Disease symptoms produced by modified *Xcc* strains containing these arTALEs were also more pronounced compared with the control (**Fig. 1**). Together, these results provided evidence that *BoIAA7c*, *BoSWEET13*, and *BoSWEET14* contribute to black rot virulence in cauliflower and function as *S* genes. To explore any synergistic interactions, the authors co-inoculated different combinations of arTALEs. They found that when all 3 genes were simultaneously coexpressed, they had an additive effect on disease severity and symptoms were even more severe than Tal12a strains alone.

**Figure 1:**
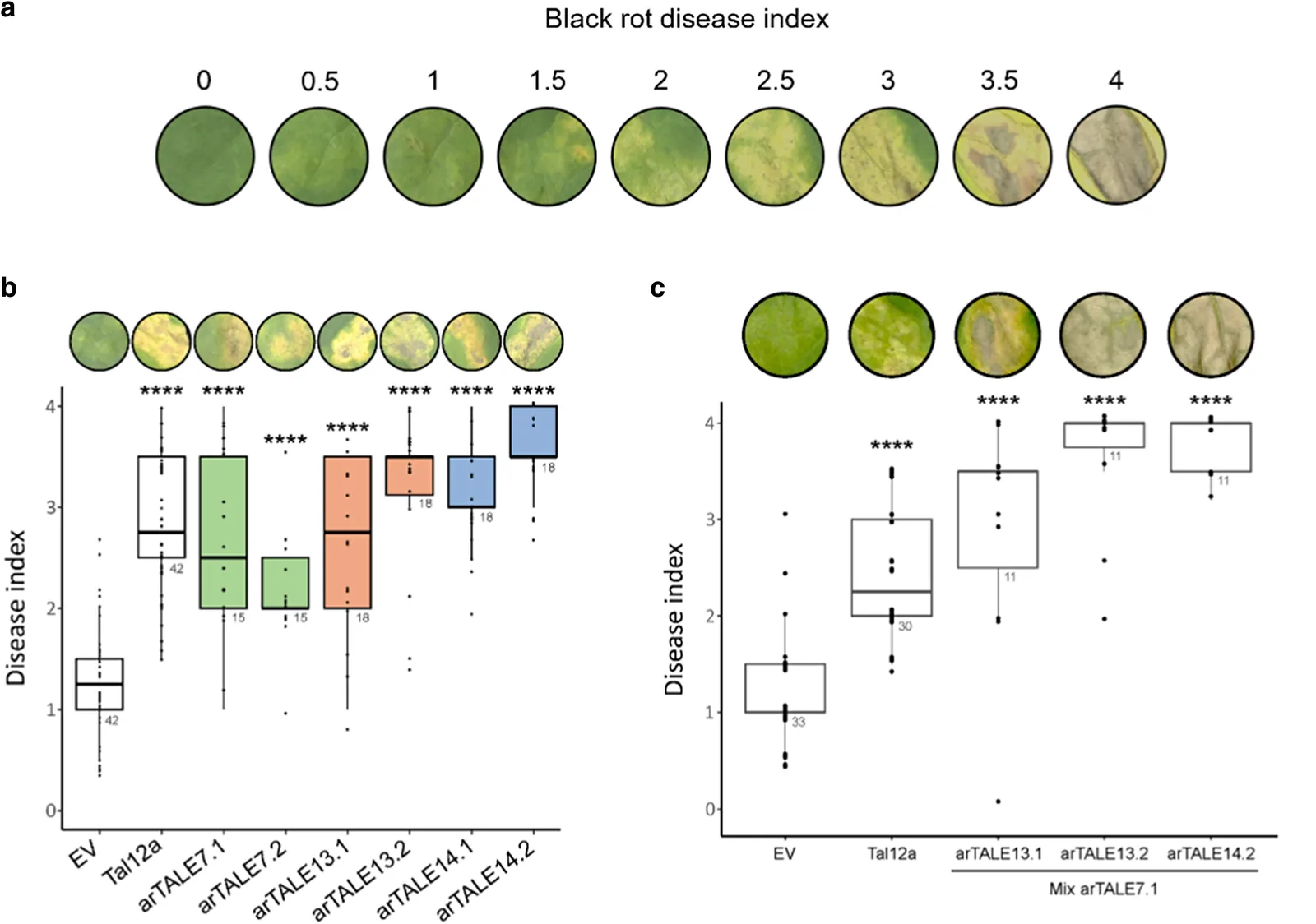
arTALES targeting *BoIAA7c*, *BoSWEET13*, and *BoSWEET14* induce black rot disease development. a) Black rot disease index ranging from 0 to 4. b) Disease index and symptoms for different arTALEs targeting the promoter regions of *BoIAA7c* (green), *BoSWEET13* (orange), and *BoSWEET14* (blue) compared with Tal12a (white). c) Disease index and symptoms for co-inoculations of different arTALE combinations. Adapted from Charleux et al. (2026) using components from Figures 6 and 7 and Figure S3. ****, *P* < 0.001. EV, empty vector.

Previous research on the virulence mechanisms of black rot in cauliflower has focused almost exclusively on the pathogen and the effectors that it injects into the host. Charleux et al (2026) identified, for the first time, *S* genes in cauliflower that are targeted by *Xcc* and that contribute to virulence. Not only does this research provide an enhanced understanding of the molecular mechanisms underlying *Xcc* pathogenesis, but the identified *S* genes serve as potential mutation targets to disrupt infection. For example, the EME sequence for *BoIAA7c* can be modified to prevent TALE binding and upregulation. Because Tal12a was found to indirectly regulate *BoSWEET13* and *BoSWEET14*, complete functional knockout of these genes may be necessary to prevent them contributing to black rot infection. Of course, the side effects of this knockout, in terms of important agronomic traits, will need to be carefully considered. These *S* genes can also be explored in different *B. oleracea* morphotypes and other *Brassica* crops as an alternative avenue to improve resistance to black rot disease.

## Recent related articles in *Plant Physiology*

Chen et al (2026) analyzed a diverse set of 70 *Xcc* strains, evaluated their pathogenicity in different *Brassica* accessions, and identified *Xop* effectors. They showed that several of these *Xop* effectors contribute to virulence in *Brassicas*, whereas TALEs were found to be dispensable in the Chinese *Xcc* strains tested.

Yang et al (2026) investigated the *Xanthomonas* effector XopAG and described how it promotes MeHFP26 degradation to suppress flavonoid biosynthesis in cassava, which weakened immunity to *X. phaseoli* pv. *manihotis*.

## Data Availability

No new data were generated or analyzed in support of this article.
